# Spectroscopic Technique-Based Comparative Investigation on the Interaction of Theaflavins with Native and Glycated Human Serum Albumin

**DOI:** 10.3390/molecules24173171

**Published:** 2019-08-31

**Authors:** Jinhui Xu, Mengyuan Wang, Yizhe Zheng, Lin Tang

**Affiliations:** Key Laboratory of Food Nutrition and Safety of SDNU, Provincial Key Laboratory of Animal Resistant Biology, College of Life Science, Shandong Normal University, Jinan 250014, China

**Keywords:** theaflavin, human serum albumin, glycated human serum albumin

## Abstract

Theaflavin is a kind of multi-pharmacological and health beneficial black tea factor. The aim of this study is to investigate the mechanisms by which theaflavin interacts with glycosylated and non-glycosylated serum albumins and compares their binding properties. Fluorescence and ultraviolet spectra indicated that theaflavin interacted with native and glycated human serum albumin through a static quenching mechanism and had a higher degree of quenching of human serum albumin. The thermodynamic parameters revealed that the combinations of theaflavin with native and glycated human serum albumin were a spontaneous endothermic reaction, and the hydrophobic force was a major driving force in the interaction process. Zeta potential, particle size, synchronous fluorescence, three-dimensional fluorescence spectroscopy and circular dichroism further clarified the effect of theaflavin on the conformation of human serum albumin structure were more pronounced. In addition, site competition experiments and molecular docking technique confirmed that the binding sites of theaflavin on both native and glycated human serum albumin were bound at site II. This study had investigated the effects of glycation on the binding of HSA with polyphenols and the potential nutriology significance of these effects.

## 1. Introduction

**Figure 1:**
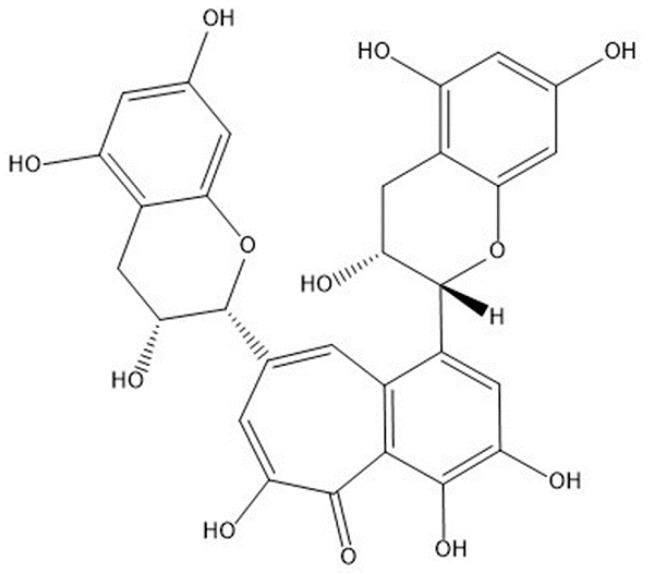
Chemical structure of theaflavin (TF).

Black tea is not only a popular drink on a global scale, but also an antioxidant available in daily life [1]. The main component of black tea is theaflavin, which is formed by oxidative condensation of polyphenols and their derivatives, soluble in organic solvents such as ethanol [2]. Theaflavin plays a decisive role in the color, aroma and quality of black tea, and also has good medical and health care functions [3]. Besides behaving good free radical scavenging properties, theaflavins also have anti-oxidation, anti-mutagenic, hypolipidemic, anti-inflammatory, anti-tumor and anti-viral effects; therefore, it attracts high hot spot in medical research [4,5,6]. In addition, the four main monomers of theaflavins are theaflavin (TF), theaflavin-3-gallate (TF2A) and theaflavin-3′-gallate (TF2B), and theaflavin-3,3′-digallate (TF3) [7]. In this study, we will use the most commonly used ligand theaflavin (TF), from previous studies. The chemical structure is shown in Figure 1.

In recent years, it has been very popular for plant extracts to be used clinically to study their physiological functions in vivo [8]. There are a large number of medical experimental studies to prove the mechanism of action of theaflavins on blood sugar and blood lipids. Skrzypczak-Jankun et al. evaluated the role of theaflavins in slowing the progression of Alzheimer’s disease or obesity through the PAI-1 dependent pathway [9]. Braud et al. showed that theaflavins protect rats from hepatic steatosis induced by a high-fat sucrose diet through their anti-lipogenic effects [10]. Takemoto et al. found that theaflavins can effectively inhibit the increase of blood glucose in hyperglycemic mice through mouse drug administration experiments [11].

Human serum albumin (HSA) is the most important transport and storage protein in the human blood circulatory system. It consists of three homologous domains that are aggregated into heart-shaped molecules [12]. Each domain is the product of two subdomains with a common structural motif, and the major region of ligand binding to human serum proteins is at Site I or Site II [13,14]. HSA contains 585 amino acid residues in which a single HSA molecule contains only one tryptophan residue (Trp214) and the major fluorescent chromophores are tryptophan and tyrosine [15]. Glycated serum protein is a kind of macromolecular ketoamine structure formed by nonenzymatic glycosylation reaction (namely glycation reactions) between serum glucose and an amino group at the N-terminus of a serum protein molecule [16]. Approximately 6% to 13% of the albumin in normal human serum is modified by nonenzymatic glycosylation, with this amount increasing by up to 20–30% in diabetic patients [16]. There are reports that the binding of HSA to some drugs can be affected by modifications resulting from glycation [17]. Although there are many studies on the combination of theaflavins with other serum proteins, there is currently no comparison of the combination of theaflavins and glycated serum proteins. In view of this, it is important to study the binding mechanism of theaflavin to human serum albumin, but it is also essential to study its binding mechanism with glycated human serum albumin.

Drug binding to serum albumin is an important determinant for their biological efficacy. In the human body, bioavailability is defined as the substances obtained from ingested materials reaching the circulatory system for further delivery into disposal sites, so that the beneficial compounds are biologically available for exerting healthy functions. Therefore, it is necessary to understand the level of binding of theaflavins with serum albumin, which will directly correlate with the bioavailability of theaflavins in vivo. However, to our knowledge, there are no literature reports show how the uptake of theaflavins affects HSA and glycated human serum albumin (GHSA), which act as transporters and stores. The objective of this study is to investigate the vitro binding mechanism of TF with HSA and GHSA using multispectral and molecular modeling techniques. The results could provide more detailed information to understand the delivery process of TF in vivo.

## 2. Results

### 2.1. Fluorescence and UV-Vis Spectra of HSA and GHSA after Addition of TF

**Figure 2:**
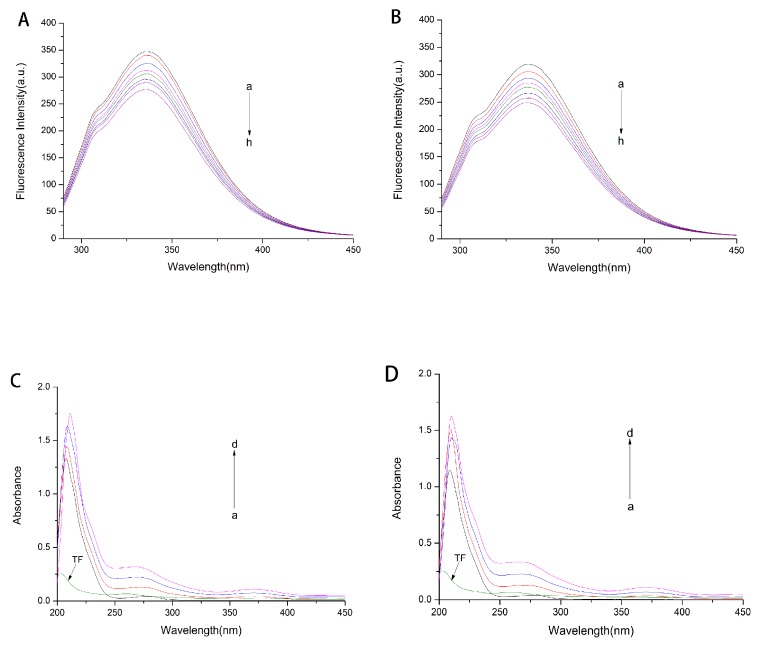
Effect of TF on fluorescence spectrum of HSA (**A**) and glycated human serum albumin (GHSA) (**B**). The fluorescence spectrum of the system is excited at 280 nm and the emission wavelength is in the wavelength range 290–450 nm. a–h: C_HSA_/C_TF_ = C_GHSA_/C_TF_ = 1:0, 1:1, 1:2, 1:3, 1:4, 1:5, 1:6 and 1:7. UV absorption spectra of HSA (**C**) and GHSA (**D**) without and with TF. a–d: C_HSA_/C_TF_ = C_GHSA_/C_TF_ = 1:0, 1:2, 1:4 and 1:6. The green line is the UV absorption spectrum of TF.

The effect of TF on the fluorescence spectra of HSA and GHSA were shown in Figure 2A,B. It could be seen that the maximum emission wavelength of HSA and GHSA was around 336 nm, while there was no emission peak for TF in this wavelength range. With the gradual addition of TF, the fluorescence intensity of HSA and GHSA showed different degrees of decline.

Ultraviolet spectroscopy is often used to determine the conformational changes of the protein itself [18].The ultraviolet absorption spectrum of the complex formed by TF and HSA/GHSA was measured, and the experimental results are shown in Figure 2C,D. It can be seen that both HSA and GHSA had two absorption peaks, which were around 205 nm and 280 nm, respectively. The absorption peak at 205 nm belongs to the skeletal peak of the protein itself, and the absorption peak at 280 nm is the absorption peak of the three fluorescent chromophores of tryptophan, tyrosine and phenylalanine in the protein [18]. As TF was gradually added to the HSA and GHSA solutions, the protein carbon skeleton and the chromophore absorption peak intensity increased. The maximum peak position of the carbon skeleton absorption peak was accompanied by a slight red shift.

### 2.2. Fluorescence Quenching Mechanism of Reaction

Fluorescence spectroscopy is commonly used to characterize the interaction of fluorescent macromolecular proteins with other small molecule ligands and to determine the quenching mechanism of complexes by fluorescence temperature change experiments [19,20]. Thence, the Stern–Volmer equation, Equation (1) was used for analysis [21]:

(1) $$F_{0}/F=1+K_{q}\tau_{0}\left[Q\right]=1+K_{\mathrm{SV}}\left[Q\right]$$

**Figure 3:**
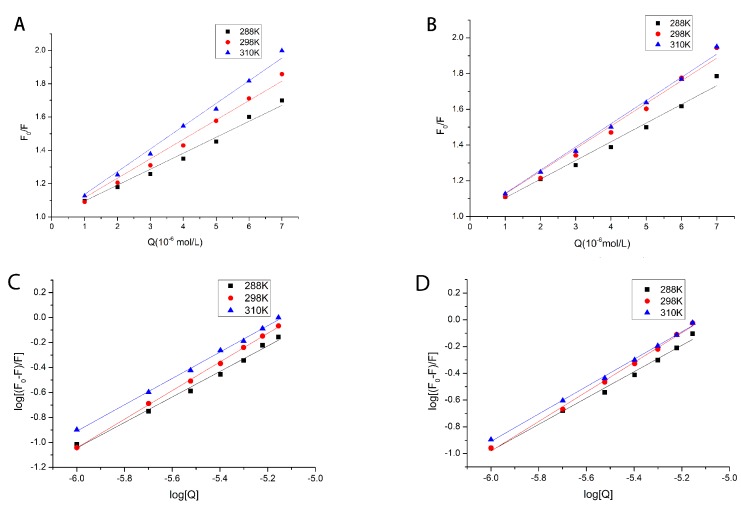
The Stern–Volmer plots for the quenching of HSA by TF (**A**) and GHSA by TF (**B**) at different temperatures (288 K, 298 K, 310 K). Plots of log [(F_0_ − F)/F] versus log [Q] for the TF–HSA systems (**C**) and TF–GHSA systems (**D**) at different temperatures.

F_0_ and F are the intensities of the protein in the absence and presence of TF, respectively. K_SV_ is the quenching constant of the Stern–Volmer equation, [Q] is the concentration of TF, K_q_ is the quenching rate constant of TF, and τ_0_ is the average fluorescence lifetime value of the macromolecule in the absence of TF, generally defaulting to 10^−8^ ns. The intercept was set to 1, and we obtained the corresponding K_SV_ value by plotting F_0_/F against [Q]. The results were shown in Figure 3A,B, and the calculation data was shown in Table 1. It could be seen in Table 1 that the K_q_ value during the reaction was much larger than the maximum diffusion collision quenching constant value (2.0 × 10^10^ L/mol/s) [21]. Therefore, the quenching mechanism existing in the process of determining the binding of TF to HSA and GHSA were a static quenching mechanism for the formation of a ground state complex [20,21].

### 2.3. Calculation and Analysis of Binding Site Number and Binding Constant

The binding constant and number of binding sites for the interaction of TF with proteins could be calculated by the double logarithmic curve, Equation (2) [22]:

(2) $$\log\left[\left(F_{0}-F\right)/F\right]=\log K_{a}+n\log\left[Q\right]$$

where K_a_ represents the binding constant and n represents the number of binding sites. By plotting log[(F_0_-F)/F] on log[Q], the corresponding K_a_ and n values could be obtained. The relevant value results were shown in Table 1 and the linear relationships were shown in Figure 3C,D. According to the data in Table 1, the n values of TF and HSA/GHSA were both around 1.

### 2.4. Thermodynamic Parameters and Properties of Binding Forces

In general, the interaction between small ligands and biomacromolecules can be calculated by formula calculations [23,24]. Therefore, in order to determine the type of non-covalent bonding interaction between TF and HSA/GHSA, we calculated the corresponding thermodynamic parameters, which were generally obtained by the van’t Hoff equation, Equations (3) and (4) [20].

(3) $$\ln K_{a}=-\Delta H/\mathrm{RT}+\Delta S/R$$

(4) ΔG=ΔH−TΔS

ΔH, ΔS, and ΔG represent the enthalpy change, entropy change, and Gibbs free energy in the interaction process, respectively; R is the gas constant (8.314 J/mol K); T is the temperature; and K_a_ is the bond constant value at the corresponding temperature. The calculation results of the thermodynamic parameters were shown in Table 1. From the data in the table, it could be seen that ΔH > 0, ΔS > 0 and ΔG < 0.

### 2.5. Determination of Amino Acid Icroenvironment Changes by Synchronous Fluorescence Spectroscopy

**Figure 4:**
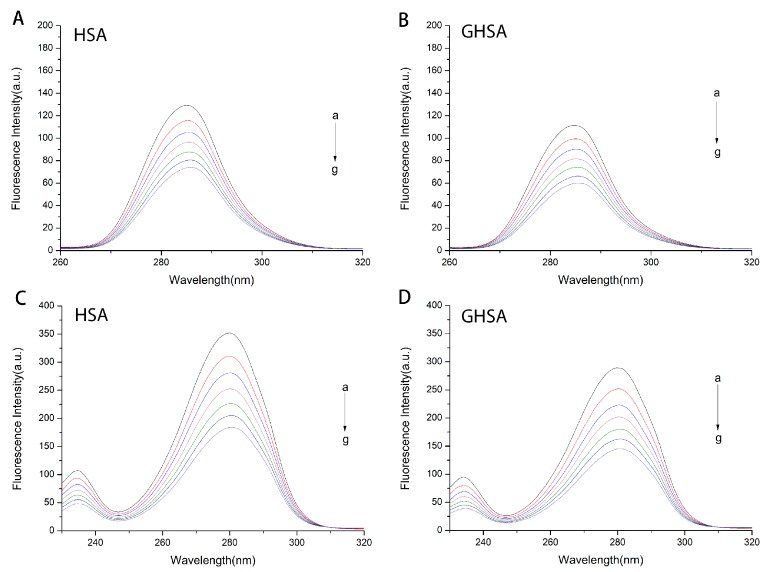
Synchronous fluorescence spectra of HSA and GHSA while varying the concentration of TF (**A**–**B**: Δλ = 15 nm, **C**–**D**: Δλ=60 nm). a–h: C_HSA_/C_TF_ = C_GHSA_/C_TF_ = 1:0, 1:1, 1:2, 1:3, 1:4, 1:5, 1:6 and 1:7.

Determination of synchronous fluorescence spectroscopy is commonly used to indicate the effect of ligands on protein conformation [25]. When the wavelength interval (Δλ) is maintained at 15 nm or 60 nm, the synchronous fluorescence provides characteristic information of the tyrosine residue or the tryptophan residue [25,26]. The experimental results were shown in Figure 4. It could be seen that the fluorescence intensity of HSA/GHSA decreased with the addition of TF, and the wavelength of the maximum fluorescence intensity had no obvious shift.

### 2.6. Study on the Change of Protein Structure in Complexes

#### 2.6.1. Zeta Potential and Particle Size

The zeta-potentials of HSA, TF–HSA, GHSA and TF–GHSA were −8.62 mV, −7.24 mV, −8.86 mV and −8.47 mV, respectively. The results of particle sizes distribution of TF–HSA/GHSA complexes were still in nano-size region. The particle sizes of HSA, TF–HSA, GHSA and TF–GHSA were 19.42 nm, 28.58 nm, 28.25 nm and 30.55 nm respectively.

#### 2.6.2. Three-Dimensional Fluorescence

Fluorescence excitation-emission matrix spectroscopy can simultaneously acquire and characterize the spectral peak information of different fluorophores of proteins [27]. When λex = λem, the peak shows a Rayleigh scattering peak; when λex = 280.0 nm and λem = 337.0 nm, the peak represents the spectral characteristics of tryptophan and tyrosine residues; and when λex = 225.0 nm and λem = 335.0 nm, the peak is the fluorescence spectrum, characteristic of the protein polypeptide skeleton, with strength related to the secondary structure of the protein [28]. Three-dimensional fluorescence measurements were performed on HSA/GHSA and the corresponding TF–HSA/GHSA system. The corresponding peak data results were shown in Table 2. It could be seen from the figure that with the addition of TF, the peak a and peak b values of HSA and GHSA had a corresponding degree of decline.

#### 2.6.3. Circular Dichroism

**Figure 5:**
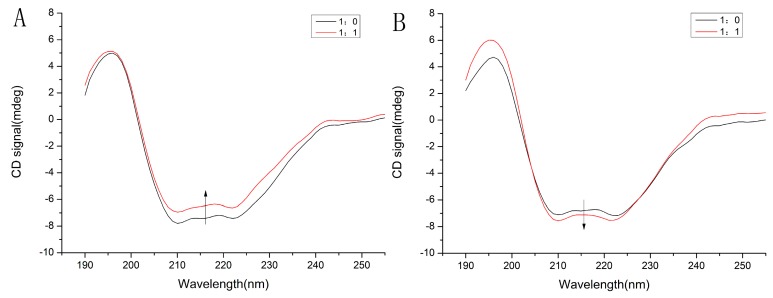
Far-UV circular dichroism (CD) spectra of HSA–TF (**A**) and GHSA–TF (**B**).

The circular dichroism can directly reflect changes in the conformational effects of the ligand on serum proteins [29]. Circular dichroic scanning spectra from 190 to 260 nm (far ultraviolet region) reflect the arrangement of protein peptide bonds and are used to calculate the ratio of protein secondary structures, namely α-helix, antiparallel, parallel, β-turn and random coil [29]. The results were shown in Figure 5A,B, and the corresponding conformational change data was shown in Table 3. Proteins have many chromophores that produce circular dichroism (CD) signals. It could be seen from Figure 5 that the α-helix exhibits strong negative absorption peaks at 208 nm and 222 nm. The addition of TF resulted in a decrease in the α-helix of HSA, while the random coil and the antiparallel, parallel and β-turn increased. In contrast, the addition of TF resulted in an increase in the α-helix of GHSA, while the random coil and the antiparallel, parallel and the β-turn showed a decreasing trend.

### 2.7. Site Marker Competition Experiments

**Figure 6:**
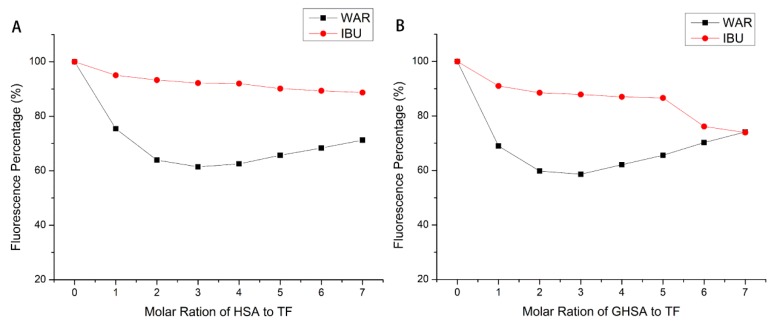
The effect of the site marker on the characteristic fluorescence of HSA (**A**) and GHSA (**B**) at the wavelength of maximum fluorescence intensity.

The binding position of TF on HSA/GHSA could be determined by site competition experiments. Studies showed that warfarin could specifically bind to serum protein at site I, while ibuprofen could specifically bind to site II [30,31,32,33]. Therefore, warfarin and ibuprofen were selected as probes for site competition, and fluorescence experiments were conducted to explore the binding mechanism [34]. By processing the fluorescence data, Figure 6 was obtained. It was seen that in the HSA/GHSA system in which TF was present; the change in fluorescence intensity caused by the addition of ibuprofen was much smaller than that of warfarin, and even tended to be unchanged. Thence, it could be confirmed that TF may share a binding site with ibuprofen.

### 2.8. Molecular Docking Experiment

**Figure 7:**
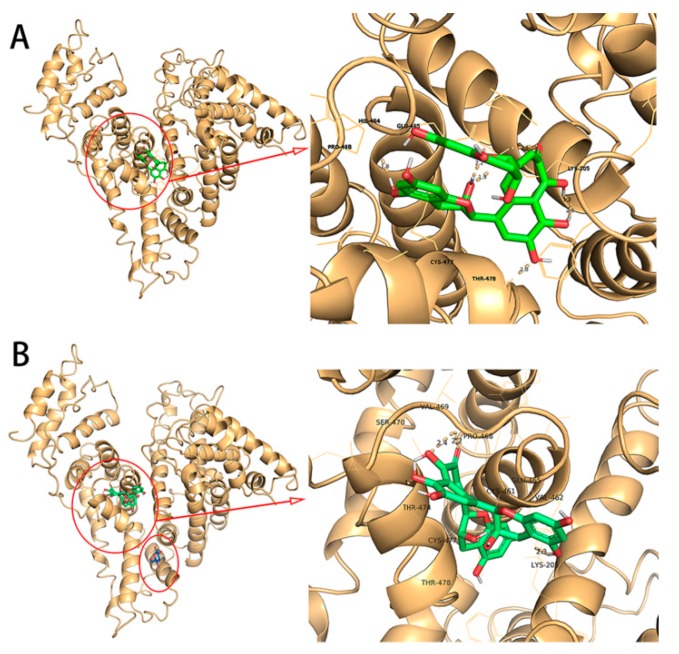
**A**: Left: The combined orientation map of the lowest docking energy conformation of TF on HSA. Right: a detailed view of the binding mechanism of TF at position 1 and the amino acid residues acting at the binding site. **B**: Left: Binding orientation map of the lowest docking energy conformation of glucose on HSA. Right: A detailed view of the amino acid residues acting on the binding site of the glucose molecule to the HSA.

The binding mode of TF and HSA/GHSA was predicted by computer simulation of molecular docking techniques. The specific results of the combination were shown in Figure 7. The figure showed the combination of TF and HSA at the lowest binding energy, and the stability of the complex in this mode was stronger compared to the other modes. The same was true for the molecular docking results of TF and GHSA. As could be seen from the graph of Figure 7A, TF was bound to subdomain IIIA of site II of the HSA [35]. In the docked conformation enlargement, it could be seen that the amino acid residues surrounding the ligand binding site were PRO 468, CYS 477, THR 478, LYS 205, HIS 464 and GLU 465. Simultaneous to the predictions of the types of amino acid residues and the binding properties at the lowest binding energies, the software also predicted that the TF–HSA system had five hydrogen bond formations, including THR 478 (1 hydrogen bond), LYS 205 (3 hydrogen bond) and GLU 465 (1 hydrogen bond). We also found through the docking results of Figure 7B that the amino acid residues around the ligand binding site in the GHSA-TF system were VAL 469, VAL 462, PRO 468, SER 470, THR474, THR 478, LYS 205, LYS 477, CYS 461, GLU 465, and there were three hydrogen bonds at the amino acid binding site, including those involving VAL 469 (1 hydrogen bond) and THR 474 (2 hydrogen bonds).

## 3. Discussion

There are two main quenching mechanisms for ligand-induced protein fluorescence quenching. One is the dynamic quenching process caused by the random encounter between the excited fluorophore and the quenching agent (ligand) molecules. The other is a static quenching process in which a non-fluorescent ground state complex is formed between the protein and the quenching agent [19,20]. The results of fluorescence spectroscopy and UV-Vis spectroscopy clearly indicated that TF to HSA and GHSA do form a complex, and the quenching mechanisms were static quenching. Furthermore, by comparing the quenching difference at the maximum fluorescence wavelength, it concluded that the quenching intensity of TF to HSA was greater than the quenching intensity of TF to GHSA, so we speculated that the binding of TF to HSA was more intense. In addition, UV spectroscopy results further confirmed the conclusion of fluorescence experiments in which TF binds to HSA and GHSA to form a complex. It was also found that the addition of TF changed the structure and polarity of HSA and GHSA, while the hydrophobicity of the microenvironment of aromatic amino acid residues was enhanced.

Besides it could be inferred from the n value results, that the interaction was a single site binding. From the data comparison, we found that the magnitude of K_a_ was 10^5^, indicating that TF could be more moderately combined with HSA/GHSA. In addition, the K_a_ value of TF–HSA was greater than that of TF–GHSA, indicating that the ability of HSA to form a complex with TF was slightly higher, which was in line with the fluorescent conclusion made before. We speculated that it was due to the combination of HSA and glucose, which reduced the binding ability of HSA and TF. In other words, it was possible that glucose inhibited the binding of TF to HSA. Therefore, it is necessary to further verify through subsequent experiments.

In general, there are four types of interacting non-covalent bonds between the interaction of small ligands and biomacromolecules, which are hydrogen bonds, van der Waals forces, electrostatic forces, and hydrophobic forces [23]. They can be determined by Ross’s thermodynamic symbol values related to the main forces of the bonding process. When ΔH > 0 and ΔS > 0, the hydrophobic effects dominates; when ΔH < 0 and ΔS < 0, van der Waals force/hydrogen bonding is the main force at play; when ΔH < 0 and ΔS > 0, it is the electrostatic force leading [24]. By analyzing the calculation results of the thermodynamic parameters, it could be concluded that the reaction was a spontaneous endothermic reaction. In addition, ΔH > 0, ΔS > 0 inferred that the hydrophobic effect played a dominant role in the interaction process. Although the reaction mechanism of TF and HSA/GHSA was roughly the same, it could be seen from the data in Table 1 that ΔH_TF–GHSA_ < ΔH_TF–HSA_ indicated the heat absorbed by TF–GHSA during complex formation was less than that of TF–HSA. This phenomenon also verified the binding strength of TF to HSA was stronger than that of GHSA. Then, the results of the simultaneous fluorescence experiment could also be seen that the addition of TF had little effect on the hydrophobicity and polarity of the microenvironment of protein tyrosine and tryptophan residues. Moreover, the fluorescence of the tryptophan and tyrosine residues of serum proteins was continuously annihilated with the continuous addition of TF, which also confirmed that TF did form a complex with HSA/GHSA.

Since the isoelectric point of native and glycated human serum is weak, both serum proteins have a global negative charge at physiological pH [36,37]. Through experiments, we found the interaction of TF and HSA/GHSA formed new complexes which made the zeta-potential smaller than before and the particle size larger than before. Interestingly, the degree of change in the TF–GHSA system is much smaller than that in the TF–HSA system, perhaps because it was previously speculated that the effect of TF and HSA was stronger than that of GHSA. Therefore, it was necessary to further explore the effect of TF on the structure of proteins after forming complexes with HSA/GHSA.

On the one hand, the peak value change results obtained by fluorescence excitation-emission matrix spectroscopy experiments showed that the structure of HSA/GHSA changed to some extent after adding TF. On the other hand in view of the conclusions from previous fluorescence spectroscopy studies, TF and HSA/GHSA both formed a single complex, so we used a binding system with a molar ratio of 1:1 to determine the circular dichroism of the complex. The results were shown in Figure 5, and the corresponding conformational change data was shown in Table 3. Proteins had many chromophores that produce CD signals. It could be seen from Figure 5 that the α-helix exhibits strong negative absorption peaks at 208 nm and 222 nm, which are characteristic of π–π* and n–π* transitions, respectively [38]. By observing the Figure 5, it was clear that the conformational change of HSA after TF addition was different from that of GHSA. Furthermore, from the secondary structure data presented in Table 3, it was found that the addition of TF resulted in a decrease in the α-helix of HSA, while the random coil and the antiparallel, parallel and β-turn increased. It indicated that the structure of the protein was slightly loose after HSA binds to TF. We speculated that it might be due to TF destroying the hydrogen bond network structure of HSA, leading to the unfolding of the helical structure of the protein [24]. However, the addition of TF resulted in an increase in the α-helix of GHSA, while the random coil and the Antiparallel, Parallel and the β-turn showed a decreasing trend. On the whole, the effect of TF combined with serum protein on the structural changes of HSA was greater than that of GHSA. We speculate that this might be due to a large change in the conformation of HSA after binding to glucose, and thus the conformational change after binding to TF was not significant. This also proved that the binding ability of GHSA to TF was weaker than that of HSA and TF.

Finally, in order to further explore the binding nature of TF to native and glycated human serum proteins, a site competition experiment was applied to show that the binding site of TF to serum protein was confirmed at site II. It was worth noting that in Figure 6, an interesting phenomenon had occurred. When increasing the concentration of (G) HSA vs TF, the reversal of the effect (even to the point of ibuprofen activity for GHSA). This phenomenon is worthy of further exploration by us. Although binding sites of TF interacting with HSA/GHSA were all confirmed at site II, whether their specific binding conditions at sites were consistent still needed to be further confirmed by molecular docking technology. By analyzing the molecular docking results of natural and glycated human serum proteins and TF, it was possible to derive simulated docking information for their complex formation. The benzene ring of the ligand was buried in the hydrophobic fracture, and interacted with the protein lipophilic residue accumulated in the binding site by hydrophobic interaction to stabilize the docking conformation [20]. The hydrogen bonds acting on the binding site also interact with the hydrophobic interaction to stabilize the docking conformation. Chitpan et al. also used the quartz crystal microbalance with dissipation monitoring to obtain the similar conclusion that the theophylline adsorption on the surface of BSA is mainly due to the interaction between the electrostatic force and hydrogen bonding [39].

The locations for several lysines that have often reported to take part in glycation have been reported in the study by Jeanethe et al. [16]. Where K439 is near the site II, K199, K233, K276, K281, K323 are close to the site I, and the lowest binding energy site of glucose to HSA was similar to one of the locations for several lysines (K323) [16]. Therefore, we selected a glycosylated human serum protein structure that bound to subdomain IIB on HSA to interface with TF, and the docking results are shown in Figure 7B. The small molecule TF bound to the hydrophobic cavity of subdomain IIIA of site II, and did not conflict with the glucose binding site on HSA subdomain IIB, indicating that GHSA could also combine well with TF to form a complex. That was also consistent with the conclusion that our previous site marker competition experiment. However, the reduction of hydrogen bonding during the binding process may be one of the reasons for the binding ability of GHSA to TF was weaker than that of HSA. Elaheh et al. also studied the binding of metformin to HSA/GHSA; the results also showed that glycosylation might destroy a binding site and reduce the ability to bind to the ligand [40]. Therefore, the molecular docking results more clearly verify the accuracy of the previous experiments comparing the strength of TF binding to HSA/GHSA.

## 4. Materials and Method

### 4.1. Materials and Equipment

Human serum albumin (A1653) and Albumin, glycated human (A8301) with a purity of 96% were purchased from Sigma-Aldrich (St. Louis, MO, USA). Theaflavins with purities of 98% were purchased from Biyu Bio-technology (Shanghai, China) Co, Ltd. Phosphate buffer saline (PBS, 0.05 M, pH 7.4) was used to dissolve protein in the experiment. The other reagents used in the experiment were analytically pure, and the water used in the experiment was ultrapure water. All samples were not further purified during the subsequent experiments.

The UV absorption spectrum was measured using a Thermo Scientific NanoDrop 2000C UV-Vis spectrometer (Thermo Scientific, Wilmington, DE, UK) equipped with a 1 cm path length quartz cell. All fluorescence data obtained from the experiments were recorded using a F7000 fluorescence spectrometer (Hitachi, Ltd., Tokyo, Japan) equipped with a 1 cm path length quartz cell. The pH of solutions was measured by a pH meter (FE28; METTLER TOLEDO Instruments (Shanghai, China) Co., Ltd.). CD spectral data of the protein solution was obtained using a CD spectrometer (Bio-Logic, 2400304, Seyssinet-Pariset, Rhone-Alpes, France) equipped with a 1 mm path length cuvette.

### 4.2. Sample Preparation

In this experiment, a stock solution of HSA and GHSA at a concentration of 5 μM was prepared in PBS buffer, and diluted with PBS to a working solution at a concentration of 1 μM. Anhydrous ethanol was used to dissolve TF to obtain a stock solution having a concentration of 1 mM. All stock solutions were stored in a refrigerator at 4 °C after preparation.

### 4.3. Fluorescence Quenching Measurements

During steady-state fluorescence measurement, a TF solution with a concentration of 1 mM was continuously titrated to a 3 mL portion of protein sample (1 μM) in quartz cuvette to get a final concentration of 7 μM. The slit width of the excited and emitted light at the time of the experiment was set to 5 nm, and the experiment was carried out at three temperatures of 288 K, 298 K and 310 K, respectively. In the simultaneous fluorescence experiment, the tyrosine residue was measured in the range of 260 nm to 320 nm, and the tryptophan residue was measured in the range of 245 nm to 320 nm. The measurement conditions of GHSA were the same as those of HSA, and all of the simultaneous fluorescence experiments were carried out at a temperature of 310 K and pH 7.4. In the fluorescence excitation emission matrix spectroscopy experiment, the parameters of the wavelength were set as follows: The excitation wavelength was from 250 nm to 350 nm; the emission wavelength was collected from 200 nm to 500 nm; and the wavelength interval was 5 nm.

### 4.4. UV-Visible Absorption Spectroscopy

3 mL of a pure protein solution having a concentration of 1 μM was added to a cuvette having a path length of 1 cm, and TF having a concentration of 1 mM was sequentially titrated to get a final concentration of 7 μM. The sample solution was mixed to be tested uniformly and scanned in the spectral range of 190 nm to 840 nm.

### 4.5. Zeta Potential and Particle Size

The zeta potential and average particle sizes of HSA/GHSA and TF–HSA/GHSA were determined by using Zetasizer Nano-ZS90 (Malvern Instruments, Worcestershire, WestMidlands, UK). The solution system of HSA/GHSA (1 mg/mL) and TF–HSA/GHSA was placed in the test vessel to test the zeta-potential and particle size respectively. The molar ratio of TF to HSA/GHSA in the mixed system was 1:1.

### 4.6. Circular Dichroism

1 mL of pure HSA and GHSA (1 μM) were mixed with TF (1 mM) to prepare a mixture at a concentration of 1 μM. A 1 mm path length cuvette was used with a scan range of 190 nm to 260 nm, and experimental data were obtained. The CDNN software was used to process the experimental data to obtain the corresponding specific gravity of each component of the secondary structure of the protein.

### 4.7. Site Marker Competitive Experiments

In the site competition experiment, two classical site probes, warfarin and ibuprofen, were selected as markers for site I and site II, respectively [41,42]. HSA/GHSA (1μM) and TF (1mM) were mixed at a molar ratio of 1:2 (pH 7.4). Then, the probe (1mM) was gradually added to HSA-theaflavin and GHSA-theaflavin to get a final concentration of 7 mM [42]. Binding studies between theaflavins and HSA/GHSA were performed using fluorescence titration. At an excitation wavelength of 280 nm, the fluorescence quenching data was recorded in the range 280–450 nm. The experiment was carried out at room temperature.

### 4.8. Molecular Docking

The simulated interaction between TF and HSA was demonstrated on a Windows 7 computer using the popular AutoDock Tool software and the AutoDock 4.2. Program (The Scripps Research Institute, La jolla, San Diego, California, USA). Molecular models of TF (MI ID: 114777) and glucose (MI ID: 64689) were downloaded at The PubChem Project and formatted using the ChemDraw tool. The available HSA crystal structure (PDB ID: 1UOR) was downloaded from the RCSB protein database and used as a receptor protein. Before the docking began, all water molecules were removed while adding polar hydrogen and Kollman charges. To explore all potential binding sites, we set enough grids (107 Å, 127 Å, 127 Å and 0.375 spacing along the x, y and z dimensions) to cover the entire protein molecule. The Lamarckian genetic algorithm was chosen to set the search parameters. The conformation with the lowest energy was chosen to further analyze the binding position of the TF and the residual microenvironment.

## 5. Conclusions

In this work, multi-spectral and molecular docking techniques were used to investigate the interaction mechanism of TF on HSA and GHSA, in order to compare the binding properties of glycosylated and non-glycosylated serum albumin to TF. Fluorescence and UV spectroscopy were used to determine that the fluorescence of HSA/GHSA was statically quenched by TF to form a complex, and the fluorescence quenching of TF by HSA was higher than that of GHSA. The binding constant and binding site number of TF interacting with HSA was greater than the corresponding value of the TF and GHSA interaction at the same temperature. Thermodynamic parameters determined that the interactions were spontaneous endothermic reactions. The hydrophobic interaction was dominated in the process of binding, further proved the hydrogen bonds which presented at the binding site also helped to strengthen the stability of the complex system. In addition, ΔH_TF–HAS_ > ΔH_TF–GHSA_ indicated that the binding strength of TF to HSA was stronger than GHSA. Three-dimensional fluorescence and CD spectroscopy demonstrated that binding of TF caused a change in the conformation of serum proteins and had a more pronounced effect on the conformational changes of HSA. Site competition experiments and molecular docking techniques determined that the binding site of TF on HSA/GHSA were on subdomain IIIA of site II and did not conflict with the glucose binding site on HSA subdomain IIB, which indicated that GHSA could also combine well with TF to form a complex.

In conclusion, although our results suggested that TF could bind to native and glycated human serum proteins to form a complex, the binding of TF to HSA was stronger. This suggests that in the environment with glycated human serum albumin as a carrier, the free form of theaflavin may be more prevalent in the environment than the human serum albumin. This work provides some valuable data for the distribution and transportation of TF in vivo, and is helpful for pharmaceutical and pharmacokinetic studies on TF.

## Figures and Tables

**Table 1:** Quenching constants, binding constants and thermodynamic parameters of the interaction between TF and HSA/GHSA at different temperatures.

Compound	T(K)	K_sv_ (10^5^Lmol^−1^)	K_q_ (10^13^Lmol^−1^s^−1^)	R^2^	K_a_ (10^5^Lmol^−1^)	n	R^2^	ΔH (KJmol^−1^)	ΔS (Jmol^−1^K^−1^)	ΔG (KJmol^−1^)
HAS + TF	288	0.9568	0.9568	0.9996	1.2191	1.0218	0.9909	123.0949	500.8975	−21.1639
	298	1.1647	1.1647	0.9995	6.8431	1.1464	0.9991			−26.1728
	310	1.3631	1.3631	0.9997	2.6491	1.0554	0.9978			−32.1836
GHSA + TF	288	1.0463	1.0463	0.9996	0.8563	0.9853	0.9907	118.561	482.6962	−20.4555
	298	1.2669	1.2669	0.9993	4.5107	1.1059	0.9964			−25.2825
	310	1.2986	1.2986	0.9998	1.7229	1.0242	0.9979			−31.0748

R^2^ is the correlation coefficient.

**Table 2:** Three-dimensional fluorescence spectral characteristics of HSA/GHSA and TF–HSA/GHSA systems.

Compound	Peak a		Peak b	
	Peak Position	Intensity F0	Peak Position	Intensity F0
	λex/λem (nm/nm)		λex/λem (nm/nm)	
HSA	225/335	266.9	280/337	239.5
HSA-TF	225/335	243.6	280/337	226.5
GHSA	225/339	267.7	280/339	238.7
GHSA-TF	225/337	237.4	280/338	217.8

**Table 3:** Secondary structure assignment of various conformational states of HSA/GHSA in the presence of TF.

Compound	α-Helix (%)	Antiparallel (%)	Parallel (%)	β-Turn (%)	Random Coil (%)
HSA	41.5	6.3	7.0	15.4	28.0
HSA-TF	37.0	7.1	8.0	16.1	30.8
GHSA	38.6	6.8	7.6	15.8	29.8
GHSA-TF	41.5	6.3	7.0	15.4	28.0

## Abbreviations

CD	Circular Dichroism
GHSA	glycated human serum albumin
HSA	human serum albumin
TF	theaflavin
UV-Vis	ultraviolet visible
